# Role of auxin homeostasis and response in nitrogen limitation and dark stimulation of adventitious root formation in petunia cuttings

**DOI:** 10.1093/aob/mcz095

**Published:** 2019-06-10

**Authors:** Huaiyu Yang, Yvonne Klopotek, Mohammad R Hajirezaei, Siegfried Zerche, Philipp Franken, Uwe Druege

**Affiliations:** 1 Leibniz Institute of Vegetable and Ornamental Crops (IGZ), Erfurt, Germany; 2 Leibniz Institute of Plant Genetics and Crop Plant Research, Stadt Seeland, Germany

**Keywords:** *Petunia hybrida*, adventitious root, root development, dark, light, nitrogen, plant hormones, polar auxin transport (PAT), auxin signalling, Aux/IAA protein, auxin response factor (ARF), small auxin-up RNA (SAUR)

## Abstract

**Background and Aims:**

Adventitious root (AR) formation in *Petunia hybrida* is inhibited by low nitrogen fertilization of stock plants but promoted by dark incubation of cuttings before planting. We investigated whether the plant hormone auxin is involved in nitrogen- and dark-mediated AR formation.

**Methods:**

Concentrations of indole-3-acetic acid (IAA) and RNA accumulation of genes controlling auxin homeostasis and function were monitored in the stem base in response to high versus low nitrogen supply to stock plants and to temporal dark vs. light exposure of cuttings by use of GC-MS/MS, a petunia-specific microarray and quantitative RT-PCR. Auxin source capacity, polar auxin transport in cuttings and auxin concentration in the rooting zone were manipulated to investigate the functional contribution of auxin homeostasis and response to the effects of nitrogen fertilization and dark exposure on rooting.

**Key Results:**

The nitrogen content of cuttings had only a marginal effect on IAA concentration in the stem base. Dark incubation enhanced the accumulation of IAA in the stem base during AR induction independent of nitrogen level. Early IAA accumulation in the dark depended on the upper shoot as an auxin source and was enhanced after apical IAA supply. Dark exposure stimulated RNA accumulation of auxin-related genes. In particular, expression of *Ph-PIN1* and of genes controlling auxin signalling, including *Ph-IAA14*, *Ph-ARF8*, *Ph-ARF10* and *Ph-SAUR14*, was enhanced, while the latter four were repressed in nitrogen-limited cuttings, particularly in the dark. Dark stimulation of rooting depended on polar auxin transport. Basal auxin application partially substituted the effect of dark exposure on rooting, whereas the auxin response of AR formation was strongly depressed by nitrogen limitation.

**Conclusions:**

Increased auxin delivery from the upper shoot and enhanced auxin signalling in the stem base contribute to dark-stimulated AR formation, while nitrogen limitation inhibits AR formation downstream of the auxin signal.

## INTRODUCTION

Adventitious root (AR) formation is a fundamental process of plant development by which cells of non-root tissues generate a new root (Steffens and Rasmussen, 2016). Excision-induced AR formation in shoot tip cuttings is of great importance for clonal propagation of many forestry and horticultural crops including petunia (*Petunia hybrida*). After excision, distinct AR source cells near the wound site of the stem base undergo a reprogramming, ultimately leading to the generation of a new root system. If the AR source cells are not competent to form a root, this reprogramming involves dedifferentiation of cells to gain root competence first, before fate-conversion to AR founder cells can be initiated by a root-inducing signal (Druege *et al.*, 2019). This induction phase is followed by the initiation of new cell clusters that successively develop into globular AR meristems, dome-shaped AR primordia and the complete root body connected to the vascular cylinder of the stem, finally followed by root emergence.

Among the endogenous factors controlling AR formation in cuttings, plant hormones have important functions with an outstanding role of auxin as a central player in hormonal crosstalk, although knowledge of the underlying processes remains fragmentary (da Costa *et al.*, 2013; Pacurar *et al.*, 2014). Indole-3-acetic acid (IAA) is the most important physiologically active auxin, and not only participates in a multitude of developmental processes but also in responses to environmental cues. It is distributed in the plant body by a fast non-directional stream in the phloem along with photosynthetic assimilates, while the formation of local IAA maxima relies on directional, cell-to-cell transport facilitated by transport proteins localized in the plasma membranes (Adamowski and Friml, 2015). Among them, PIN proteins have a prominent role as auxin efflux facilitators in directing polar auxin transport (PAT) (Adamowski and Friml, 2015).

After establishing petunia as a model system for analysis of adventitious rooting in cuttings (Ahkami *et al.*, 2009), several studies have investigated the role of plant hormones during standard cultivation of cuttings under diurnal light. Ahkami *et al.* (2013) provided evidence that a peak of IAA in the stem base during the induction phase depends on PAT and is essential for subsequent development of ARs. In addition, it contributes to the early rise in the local activity of apoplastic invertase that has an important function in the establishment of the new sink in the rooting zone (Ahkami *et al.*, 2009). Monitoring the transcriptome in the stem base revealed a fine-tuning of the auxin transport machinery and a complex phase-specific regulation of the auxin response cascade, including downstream transcription factors that link auxin signalling to cell division and specification (Druege *et al.*, 2014; Bombarely *et al.*, 2016). The transcriptome data and the response of the root phenotype of petunia to chemical and transgenic manipulations highlighted ethylene and jasmonic acid as additional positive regulators and cytokinins (Ck) as inhibitors of AR induction, while microarray data further support a negative role of strigolactones (reviewed by Druege and Franken, 2019).

Clonal propagation of many ornamental plants involves dark incubation of cuttings after harvest from the stock plant over a period of several days or weeks before planting, usually at reduced temperatures to slow down catabolic depletion of cuttings (Druege, 2009). Interestingly, AR formation in petunia is stimulated by dark incubation of cuttings before planting, which is based on the establishment first of new cell clusters during the dark phase and then accelerated AR differentiation during post-dark cultivation under light (Klopotek *et al.*, 2010). During the dark phase, carbohydrate concentrations decrease particularly in leaves (Klopotek *et al.*, 2010), while apoplastic invertase is activated specifically in the stem base, which correlates with the transcription levels of the cell-wall invertase gene *Ph-INVcw2* (Klopotek *et al.*, 2016). The local rise in activity of apoplastic invertase obviously enhances the sink competitiveness of the stem base against the upper shoot, so that during the post-dark phase the high photosynthetic rate powers a preferential allocation of carbon towards the developing ARs (Klopotek *et al.*, 2016).

The established general role of auxin and its function in activation of apoplastic invertase discussed above supported the hypothesis that changes in auxin homeostasis or response contribute to the dark stimulation of AR formation in petunia. Given the recent finding that nitrogen limitation, which has a complex impact on auxin homeostasis and signalling (Vidal *et al.*, 2010; Jin *et al.*, 2012; Ljung, 2013), impairs AR formation in petunia (Zerche *et al.*, 2016), the initial nitrogen content of cuttings may additionally modify the functional chain between auxin action and AR formation.

Following the auxin hypothesis further, the aim of this study was to elucidate the contribution of auxin to nitrogen limitation mediated by the fertilization of stock plants and to dark stimulation of AR formation in shoot tip cuttings of petunia. We investigated whether changes in auxin homeostasis and/or response underlie the nitrogen- and dark-mediated effects and which molecular processes might be involved. IAA and transcript levels of genes controlling auxin homeostasis and signalling were monitored in the stem base under different environmental conditions and combined with physical and chemical manipulations of auxin source capacity and PAT in cuttings and of auxin concentration in the rooting zone.

## MATERIAL AND METHODS

### Plant material and growth conditions

*Petunia hybrida* ‘Mitchell’ was grown from seeds and cultivated under glasshouse conditions as described by Klopotek *et al.* (2010). After planting in plastic pots containing a special mix of peat-based substrate without slow-release fertilizers (Einheitserde Typ ED-73 with Optifer, Patzer, Sinntal-Jossa, Germany), stock plants were established with a uniform nutrient availability, pH and osmotic properties in the peat substrate (in mg L^−1^: 167 NO_3_-N, 75 P, 133 K, pH 4.1, salt equivalent 1.83 g L^−1^ KCl). Depending on the particular experiment, stock plants were thereafter either generally cultivated with high nitrogen supply or divided into two groups receiving two contrasting levels of nitrogen fertilization. All experiments involved four replication plots per nitrogen treatment, using between 10 and 15 stock plants per replication depending on the particular experiment.

Nitrogen supply to stock plants was conducted according to the protocols described by Zerche *et al.* (2016). High nitrogen fertilization aimed at sufficient nitrogen nutrition without critical accumulation of nutrients or salt in the root zone of the stock plants, whereas low nitrogen supply aimed at a physiological limitation of nitrogen without showing N deficiency symptoms. Monitoring shoot biomass production and the chemical properties of the peat substrate, adequate nutrient solutions were prepared by combinations of NH_4_NO_3_ (9 % NO_3_-N + 9 % NH_4_-N), Ca(NO_3_)_2_ (19 % Ca + 14.5 % NO_3_-N + 1 % NH_4_-N) and the complex fertilizer Ferty Basis 1 (14 % P_2_O_5_, 38 % K_2_O, 5 % MgO, and micronutrients; Planta Düngemittel GmbH, Regenstauf, Germany) and applied weekly.

Shoot tip cuttings, consisting of the terminal shoot with four to five leaves, were excised 4 h after beginning of the photoperiod. The two fertilizing regimes of stock plants provided total N contents in the cutting dry matter of 3828 ± 114 µmol g^−1^ (54 ± 2 mg g^−1^) of high-nitrogen cuttings (*n* = 7 experiments) vs. 2770 ± 70 µmol g^−1^ (39 ± 1 mg g^−1^) of low-nitrogen cuttings (*n* = 5 experiments). With each nitrogen treatment, half the cuttings were exposed to light (Treatment: Light). They were planted in perlite Perligran A with a particle size of 0–6 mm (Knauf Perlite GmbH, Dortmund, Germany) and cultivated under diurnal light conditions in a growth chamber [temperature: 22/20 °C day/night, humidity outside the covered trays: 85/60 % day/night, light: 10 h day length, photosynthetic photon flux density (PPFD) of 100 µmol m^−2^ s^−1^ at plant level] as further described by Klopotek *et al.* (2010). The other half of the cuttings were exposed to darkness over a period of 7 d at 10 °C by incubation in non-perforated polyethylene bags in a cardboard box in a dark cabinet (Treatment: Dark). For assessment of AR formation, the dark incubated cuttings were planted thereafter and exposed to the same light conditions as the light-treated cuttings.

### Physical and chemical manipulation of cuttings

To investigate the contribution of the upper shoot of the cutting to the auxin status of the stem base, the shoot above the two basal leaves was removed from half the cuttings (treatment ‘decapitated’) immediately after excision from the stock plants. For evaluation of PAT in the lower stem, lanolin paste (Sigma-Aldrich, Taufkirchen, Germany) containing 3 or 0 mg g^−1^ IAA (Ducheva, Haarlem, The Netherlands) was applied to the apical cut surface of decapitated cuttings. To analyse the response of AR formation to auxin availability in the rooting zone, lanolin paste containing 0, 0.1, 1 or 3 mg g^−1^ IAA was applied to the basal cut surface of cuttings over a period of 3 d after planting. To study the contribution of PAT to AR formation in cuttings, cuttings were sprayed with water containing 0.025 % dimethyl sulfoxide (DMSO, Sigma-Aldrich) and 100 µm or 0 µm of *N*-1-naphthylphthalamic acid (NPA, Ducheva).

### Sampling for biochemical and molecular analysis and determination of rooting response

At specified hours or days after excision of the cuttings (hpe or dpe, respectively), their stem bases, 0.5 cm in length and representing the rooting zone, were shock-frozen in liquid N and stored at –80 °C until extraction for biochemical or molecular analysis. At specified hpe or days after planting (dpin) of other cuttings, ARs were counted and assigned to different classes of root length (ranges of 1 cm). The percentage of rooted cuttings, the number of ARs formed per planted and per rooted cutting, the mean length per AR and the total root length per planted cutting were calculated as described by Druege *et al.* (2007) and Agulló-Antón *et al.* (2011).

### Analysis of plant hormones

Concentrations of IAA in the stem base of cuttings were analysed on a Varian Saturn 2200 ion-trap mass spectrometer connected to a CP-3800 gas chromatograph (Agilent, Santa Clara, CA, USA) using (^2^H)_2_-IAA as internal calibration standard. The general extraction and clean-up protocols of samples, parameters of gas chromatography and settings of mass spectrometry of IAA are described by Ahkami *et al.* (2013).

### Microarray analysis of transcriptome in the stem base

After extraction of RNA as described by Bauerfeind *et al.* (2015), a broad analysis of transcript levels of genes related to auxin was conducted using a petunia-specific microarray first described by Breuillin *et al.* (2010). Probe synthesis, array hybridization and normalization of the whole data set were conducted by OakLabs (Hennigsdorf, Germany). Sequences of the microarray were classified into functional categories according to Ahkami *et al.* (2014) and Druege *et al.* (2014). Selected genes putatively controlling auxin homeostasis, signalling and function were further annotated by utilizing the recently published genomes of parental species of *P. hybrida* (Bombarely *et al.*, 2016).

### Quantitative RT-PCR analysis of selected genes

In an independent experiment, quantitative (q) RT-PCR was used to analyse the transcript levels of *Ph-PIN1*, *Ph-LAX2*, *Ph-GH3.6*, *Ph-GH3.10*, *Ph-IAA14*, *Ph-IAA19, Ph-ARF5*, *Ph-ARF8*, *Ph-ARF10*, *Ph-SAUR14*, *Ph-SAUR55* and *Ph-CYCB1* under the influence of nitrogen pre-conditioning (N-high vs. N-low) and light exposure of cuttings (Dark vs. Light). RNA was extracted using the RNeasy Mini Kit (Qiagen, Hilden, Germany). Following treatment with DNase I (Qiagen) and testing for DNA contamination, first-strand cDNA was transcribed using the Quantitect Reverse Transcription Kit (Qiagen) according to the manufacturer’s protocol. Gene expression was analysed on the CFX96 Real-Time-System (Bio-Rad, Munich, Germany) using SsoFast EvaGreen supermix (Bio-Rad). The results were evaluated with CFX Manager 2.1 software (Bio-Rad). Primers, which are described in Supplementary Data Table S1, were selected with Primer3Plus (Untergasser *et al.*, 2012). Melting curve analysis and agarose gel electrophoresis confirmed the specificity and quality of the PCR products. After preliminary tests according to Mallona *et al.* (2010), *Ph*-*RPS13* (*Ribosomal protein S13*) was used as a reference gene, which showed stable expression in the stem base under the experimental conditions. Relative transcript levels of time-course values were determined by the 2^−∆∆CT^ method.

### Statistics

All data are given as means and standard errors (s.e.), while numbers of biological replications (*n*) are indicated. STATISTICA 6.1 software (Statsoft, Hamburg, Germany) was used for statistical analysis of plant hormone concentrations, qRT-PCR results and rooting data at specified dates after excision or planting of the cuttings. A *t*-test at a significance level of *P* ≤ 0.05 was applied to mono-factorial designs. In the other cases, a two-factorial ANOVA was applied and differences between mean values were tested using Tukey’s honest significant difference (HSD) or Unequal N HSD test at a significance level of *P* ≤ 0.05.

The rank product running (RP) 1000 permutations (Breitling *et al.*, 2004) was used for statistical comparison of the microarray data of individual genes at specified dates between the dark- and light-exposed cuttings. In addition, ratios of expression data between dark (x) and light (y) exposed cuttings are presented as *M*-values (log_2_ (x/y)). If *M*-values were >1 or <−1 and RP values were <0.01, differences between samples were taken as significant. Similarly, from the qRT-PCR data of selected genes, the ratios of expression data (x/y) were calculated between the high-nitrogen cuttings in the dark (x1), the low-nitrogen cuttings in the light (x2) or the low-nitrogen cuttings in the dark (x3), and the high-nitrogen cuttings in the light (*y*), respectively. *M*-values (log_2_ (x/y)) were calculated to determine the responses to dark (x1), nitrogen limitation (x2) or the combination of both (x3).

## RESULTS

### Concentration of IAA in the stem base as affected by nitrogen pre-condition, dark incubation and the apical auxin source

**Fig. 1.**
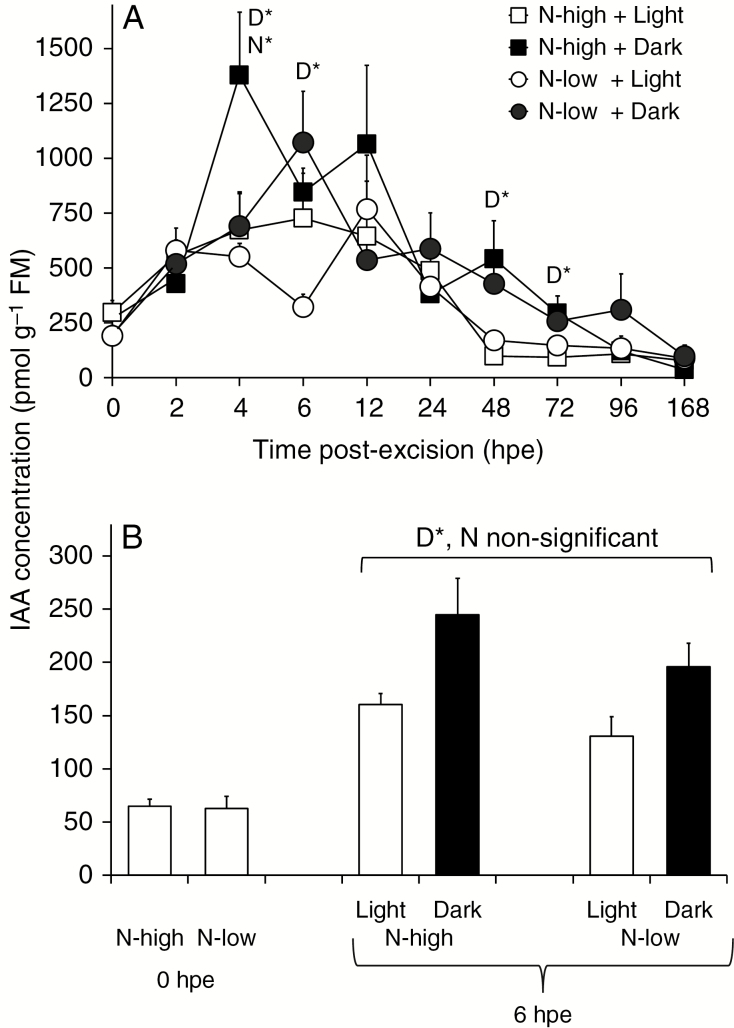
Effects of nitrogen pre-conditioning and light exposure of cuttings on the auxin status of the stem base of cuttings of *Petunia hybrida* reflected by (A) the temporal distribution of indole-3-acetic acid (IAA) concentration (first experiment) and (B) early accumulation of IAA (second experiment). Mean values and s.e. of four to eight independent biological replicates (*n*), each consisting of two cuttings. D* and N* at specified hours post-excision (hpe) indicate significant and independent effects of dark incubation and nitrogen pre-condition, respectively. ANOVA (*P* ≤ 0.05). FM, fresh mass.

To elucidate the influences of nitrogen fertilization of stock plants and dark incubation of cuttings on the auxin status of the root regenerating zone, the temporal distribution of IAA was analysed in the stem bases of high- vs. low-nitrogen cuttings at short time intervals after dark incubation and compared to cuttings planted and cultivated under light. Independent of nitrogen pre-condition, dark incubation of cuttings enhanced the early accumulation of IAA at 4 and 6 hpe and reduced the decline of the IAA peak between 48 and 72 hpe (Fig. 1A). The nitrogen content of cuttings had no effect on IAA concentration, except for a transiently lower value in low-nitrogen cuttings at 4 hpe. However, when the period from 4 to 12 hpe was analysed as a whole, only the effect of light was significant (*P* = 0.007521). The minor role of nitrogen for IAA concentrations was confirmed in the following experiment focusing on the situations at excision and 6 h thereafter. Dark incubation of both low- and high-nitrogen cuttings enhanced early IAA accumulation when compared to light exposure, while the same concentrations were measured for both nitrogen treatments (Fig. 1B).

**Fig. 2.**
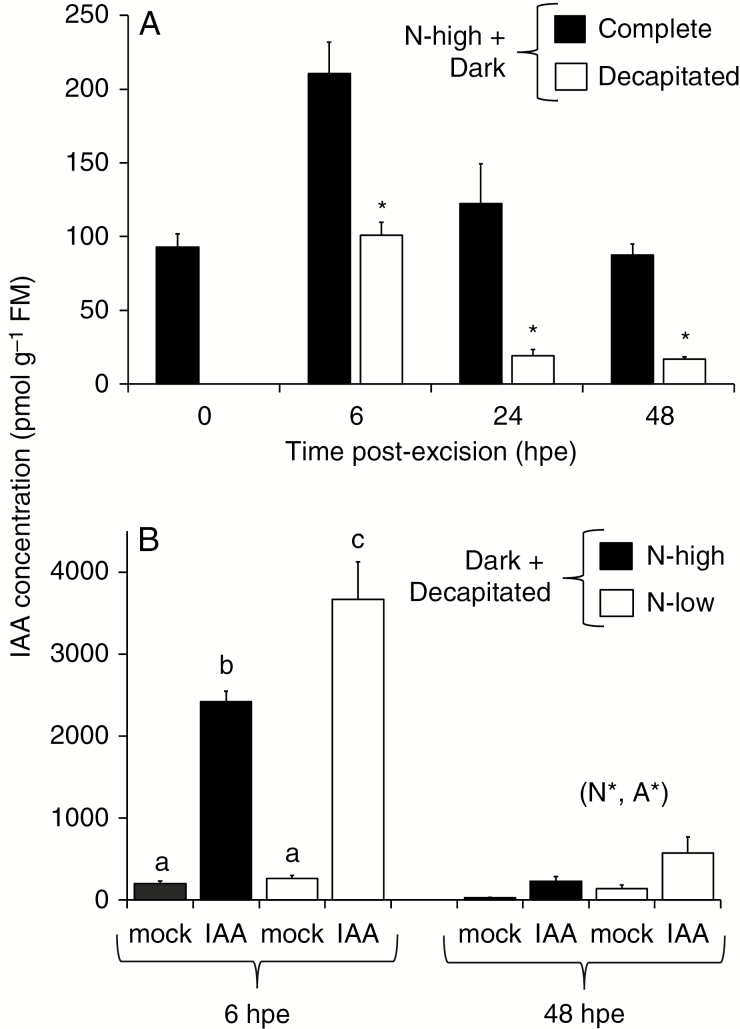
Concentration of indole-3-acetic acid (IAA) in the stem base of *Petunia hybrida* during adventitious root induction (A) in high-nitrogen cuttings in the dark as affected by removal of the upper shoot above the second oldest leaf (decapitated compared with complete cuttings), (B) in decapitated cuttings in the dark as affected by nitrogen pre-conditioning of cuttings, apical application of IAA (3 mg g^−1^ lanolin) and hours post-excision (hpe). Mean values and s.e. of five or six independent biological replicates (*n*), each consisting of two cuttings. In A, asterisks indicate a significant effect of decapitation. In B, N* and A* indicate significant effects of nitrogen pre-condition and IAA application, respectively. In case of significant interactions between the two factors, different lower-case letters indicate significant differences between the specified combinations. ANOVA, Tukey’s HSD test (*P* ≤ 0.05). FM, fresh mass.

Since the stem base of cuttings is insulated from light in the rooting substrate during cultivation, the light condition of the stem base might be similar for dark-incubated cuttings when compared to cuttings planted and cultivated under light. Results from carnation (*Dianthus caryophyllus*) indicated that the basal leaves of cuttings may provide the auxin source for adventitious rooting (Garrido *et al.*, 2002). Considering these relationships, in high-nitrogen cuttings we analysed whether removal of the upper shoot above the two basal leaves interferes with IAA accumulation in the stem base during dark incubation. The decapitation of cuttings completely prevented the early IAA accumulation until 6 hpe and caused a strong depletion of IAA until 48 hpe, falling below the initial level (Fig. 2A).

In the next experiment, we investigated the intensity of PAT in the lower decapitated cutting part when exposed to dark. When the apical cut surface of decapitated cuttings was treated with lanolin containing 3 mg g^–1^ IAA during dark exposure, the accumulation of IAA was strongly enhanced in the stem base until 6 hpe, indicating a highly efficient PAT in the stem (Fig. 2B). Although the IAA-containing lanolin remained attached to the apical cut surface of the cutting, IAA concentrations in the stem base strongly declined until 48 hpe, revealing that the entering IAA was efficiently metabolized. IAA concentrations were significantly lower in high-nitrogen cuttings at both time points (Fig. 2B).

### Response of the auxin-related transcriptome in the stem base to cutting incubation in the dark

Based on the finding that IAA levels in the stem base were strongly enhanced by dark incubation (Fig. 1), we focused on high-nitrogen cuttings and investigated the dark response of the transcriptome of auxin-related genes in the stem base using a petunia microarray. The time points of 24 and 72 hpe were analysed as important stages of gene regulation during AR induction and AR initiation under light (Druege *et al.*, 2014). Transcript levels of auxin-related genes were mostly enhanced by dark incubation of cuttings, while the general response was stronger at 72 hpe than at 24 hpe (Supplementary Data, Table S2A).

**Fig. 3.**
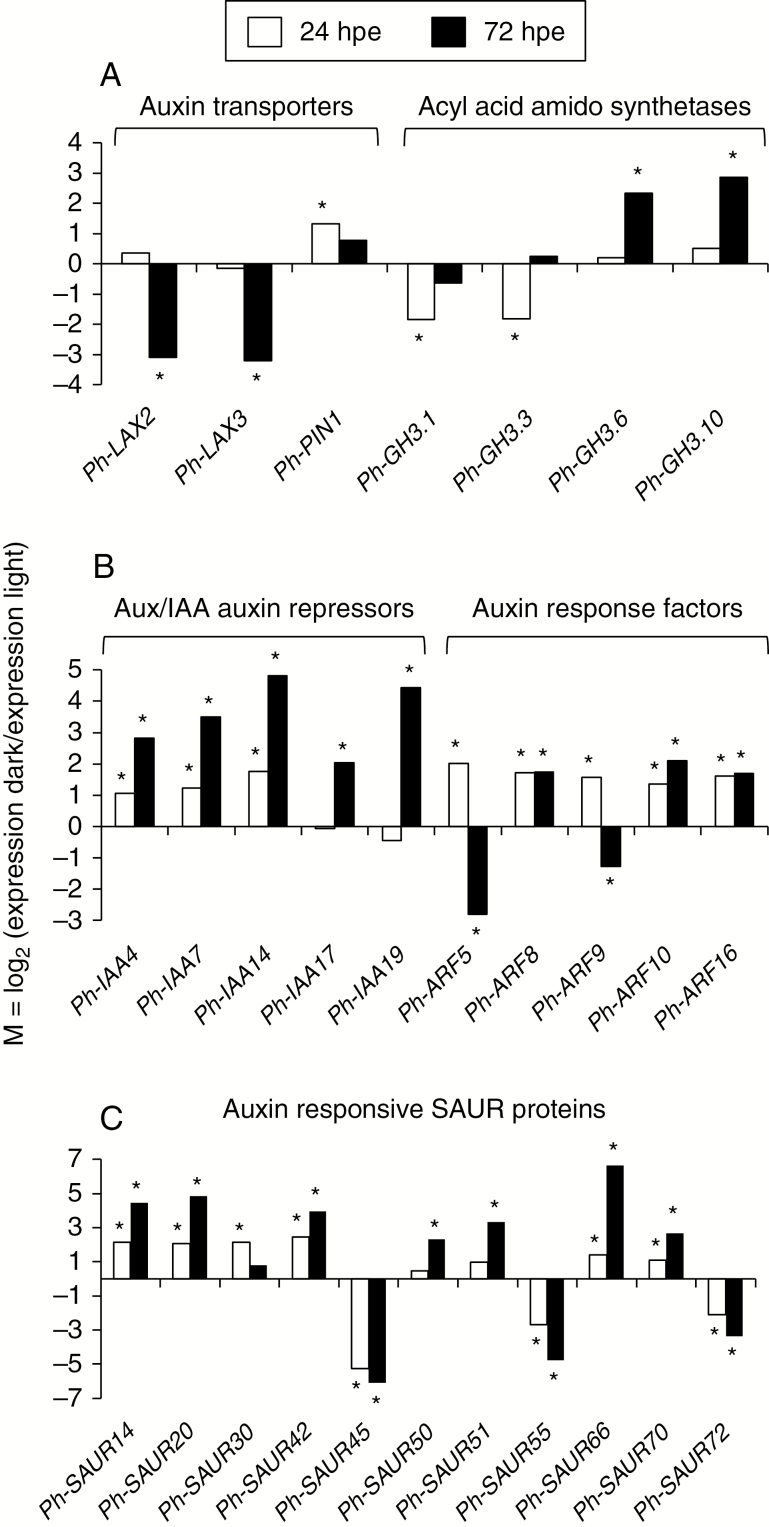
Dark response of transcript levels of genes controlling auxin homeostasis, signalling and function in the stem base of cuttings of *Petunia hybrida*, 24 and 48 h post-excision (hpe) of cuttings as revealed by microarray analysis. Genes encoding (A) LAX- and PIN-type auxin transporters and acyl acid amido synthetases (GH3 proteins), (B) Aux/IAA-type auxin repressors and auxin response factors (ARFs), (C) auxin responsive SAUR proteins. M = log_2_ of ratio (dark/light) of transcript levels of genes, *P* ≤ 0.05 (*t*-test, *n* = 4, each consisting of 8–12 cuttings).

Detailed analysis of 28 genes putatively controlling auxin homeostasis, signalling and function revealed that 17 genes were exclusively up-regulated and five genes were exclusively down-regulated by dark exposure at least at one time point (Supplementary Data Table S2B). Figure 3 illustrates the relative dark response of transcript levels of these genes encoding putative (A) auxin transporters and Gretchen Hagen 3 (GH3) proteins that function as acyl acid amido synthetases, (B) Aux/IAA-type auxin repressor proteins and auxin response factors (ARFs) as components of the auxin response machinery, and (C) Small Auxin UP RNA (SAUR) proteins as auxin responsive proteins. Two genes encoding two LAX auxin influx transporters were down-regulated after 3 d of darkness, whereas the auxin efflux transporter gene *Ph-PIN1* was up-regulated at 24 hpe (Fig. 3A). *GH3* genes showed variable dark responses among specific family members. *Ph-GH3.1* and *Ph-GH3.3* were down-regulated after 24 h, whereas *Ph-GH3.6* and *Ph-GH3.10* were up-regulated after 72 h of darkness (Fig. 3A). Interestingly, five genes encoding Aux/IAA auxin repressor proteins were up-regulated by dark exposure, with the strongest response observed for *Ph-IAA14* and *Ph-IAA19* at 72 hpe (Fig. 3B). Furthermore, transcript levels of five *ARFs* were enhanced after 24 h of darkness. Up-regulation of *Ph-ARF8*, *Ph-ARF10* and *Ph-ARF16* was maintained until 72 hpe, whereas transcript levels of *Ph-ARF5* and *Ph-ARF9* were negatively affected by dark exposure at this time point (Fig. 3B). The auxin-responsive gene family of SAUR proteins showed the broadest response to dark incubation of cuttings. Eight genes were up-regulated with the strongest increase at 72 hpe, whereas transcript levels of *Ph-SAUR45*, *Ph-SAUR55* and *Ph-SAUR72* were reduced by the same treatment (Fig. 3C). The data as a whole support the model that the greater accumulation of IAA in the dark (Fig. 1) stimulates the transcription of *ARF* genes as important positive regulators of the auxin signalling cascade, which modifies the downstream expression of *SAUR* genes.

### Influence of nitrogen pre-condition and light on transcript abundance of genes controlling auxin homeostasis and signalling

To obtain a higher temporal resolution of the expression of auxin-related genes in the stem base in response to dark incubation of cuttings and to investigate its dependency on the nitrogen pre-condition of cuttings, two different nitrogen treatments and seven time points of analysis were considered in the next experiment. Eleven genes, transcript levels of which had shown characteristic responses to dark in the microarray study, and a petunia homologue of the auxin-responsive cyclin gene *CYB1* were selected and analysed by qRT-PCR. The expression values generally confirmed the microarray study. The data revealed a dominant, mostly promotive influence of dark incubation and a less pronounced effect of low nitrogen supply while at certain time points, the transcript levels were subject to interaction between both factors (Supplementary Data Table S2C). However, dark-induced up-regulation of *Ph-PIN1* and down-regulation of *PH-LAX2* were independent of nitrogen and observed earliest after 24 hpe (Supplementary Data Table S2C), which was 20 h after IAA had started to accumulate (Fig. 1A).

**Fig. 4.**
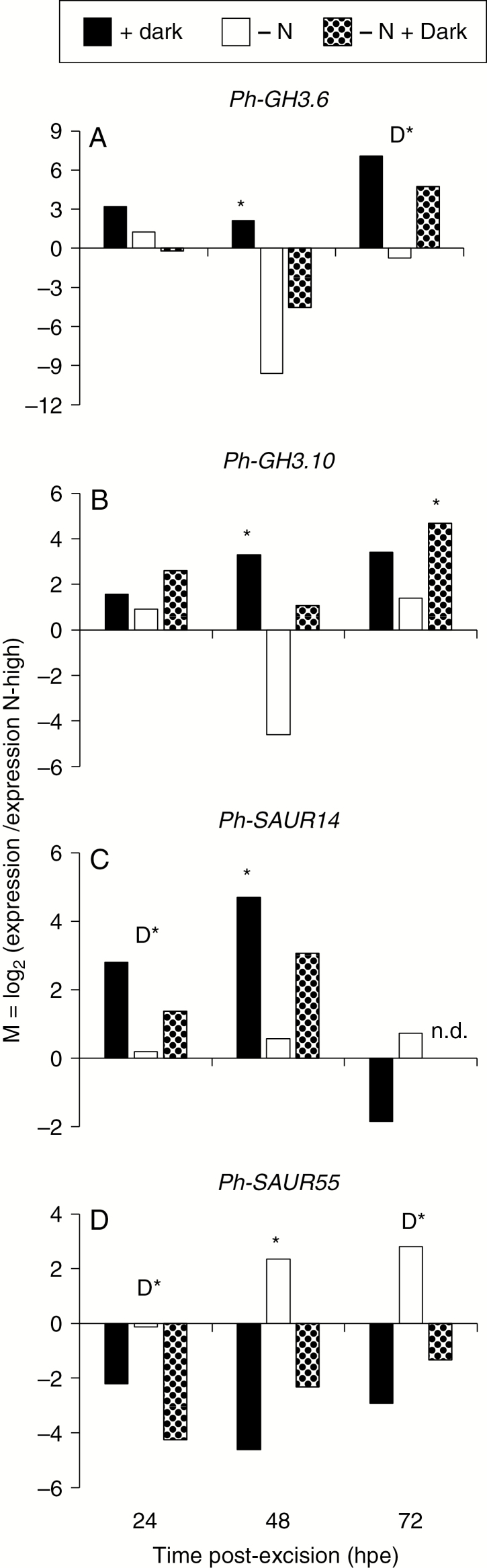
Response of transcript levels of selected genes encoding GH3 and SAUR proteins in the stem base of *Petunia hybrida* to dark incubation, nitrogen limitation or the combination of both, 24 and 72 h post-excision (hpe) of cuttings as revealed by microarray analysis. (A) *Ph-GH3.6*, (B) *Ph-GH3.10*, (C) *Ph-SAUR14* and (D) *Ph-SAUR55.* M = log_2_ (x/y) of transcript levels of genes (x1 = N-high under dark, x2 = N-low under light, x3 = N-low under dark, *y* = N-high under light). qRT-PCR. D* indicates a significant main effect of dark incubation. In case of significant interactions between nitrogen and dark incubation, asterisks indicate those treatments that were significantly different from N-high light-exposed cuttings. ANOVA, Tukey’s HSD test (*P* ≤ 0.05).

**Fig. 5.**
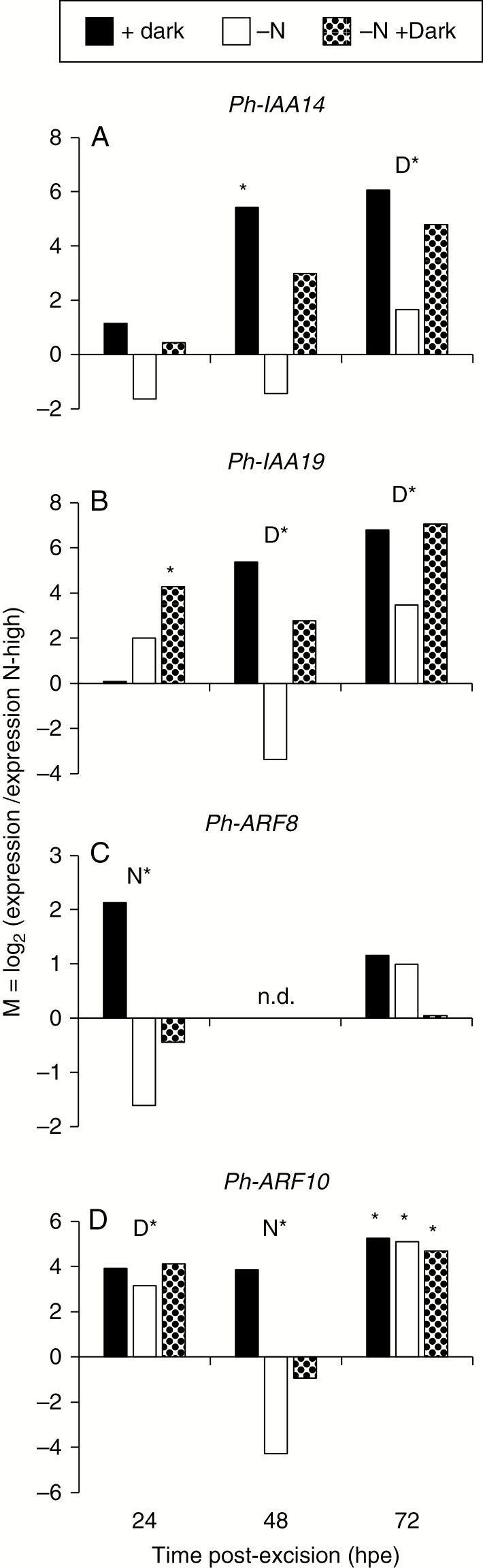
Response of transcript levels of selected genes encoding Aux/IAA auxin repressors and ARFs in the stem base of *Petunia hybrida* to dark incubation, nitrogen limitation or the combination of both between 24 and 72 h post-excision (hpe) of cuttings as revealed by qRT-PCR. (A) *Ph-IAA14*, (B) *Ph-IAA19*, (C) *Ph-ARF8* and (D) *Ph-ARF10.* N* and D* indicate significant main effects of nitrogen pre-condition or dark incubation, respectively. In case of significant interactions between nitrogen and dark incubation, asterisks mark those treatments that were significantly different from N-high light-exposed cuttings. ANOVA, Tukey’s HSD test (*P* ≤ 0.05).

For the time period between 24 and 72 hpe, the expression values of the high-nitrogen cuttings exposed to light were used as reference to calculate the relative change of expression of selected genes in response to dark exposure, to nitrogen limitation or to the combination of both. Figures 4 and 5 illustrate a dominant, mostly positive effect of dark exposure on the expression of auxin-related genes and a less frequent, mostly inhibitory role of nitrogen limitation, either by reducing transcript levels independent of dark exposure (Fig. 5C, D) or by repressing the dark response of gene expression (Figs 4 and 5A). *Ph-GH3.6* and *GH3.10* were up-regulated after 48 and 72 h of darkness, whereas at 48 hpe this response was repressed in nitrogen-limited cuttings (Fig. 4A, B). The expression analysis confirmed the dark-induced up-regulation of *Ph-IAA14* and *Ph-IAA19*. This was, however, modified by the nitrogen content of cuttings in an opposite manner between the two auxin repressor genes (Fig. 5A, B). Nitrogen limitation reduced the expression of *Ph-IAA14* at 0.5 and 6 hpe independent of light (Supplementary Data Table S2C) and inhibited the dark-induced up-regulation of the same gene at 48 hpe (Fig. 5A). By contrast, the same treatment enhanced transcription of *Ph-IAA19* at 0.5 hpe at marginal levels and provoked its dark-induced up-regulation at 24 hpe (Supplementary Data Table S2C, Fig. 5B).

The transcript levels of *Ph-ARF8* and *Ph-ARF10* were reduced in nitrogen-limited cuttings at 24 and 48 hpe, respectively, thereby preventing dark-induced up-regulation (Fig. 5C, D). Correspondingly, the dark-induced up-regulation of *Ph-SAUR14* was repressed at 48 hpe by nitrogen limitation (Fig. 4C). By contrast, transcript levels of *Ph-SAUR55* were decreased by dark exposure from 24 hpe onwards as in the microarray study but were enhanced by nitrogen limitation at 48 hpe when cuttings were cultivated under light (Fig. 4D). Transcript levels of *Ph-CYCB1* were transiently depressed by dark incubation until 72 hpe but thereafter attained the same levels until 168 hpe in the dark as under light (Supplementary Data Table S2C).

### Effect of NPA and basal IAA treatment on the dark- and nitrogen response of AR formation

Analysis of IAA concentration and the transcriptome of auxin-related genes in the stem base revealed that dark incubation of cuttings enhances auxin accumulation in the stem base. This depended on the upper shoot. Dark incubation also stimulated different molecular components of the auxin response cascade in the stem base, some of which were negatively affected by nitrogen limitation. In the two following experiments we analysed whether the dark response of AR formation in petunia is dependent on PAT and can be substituted by auxin application to light-treated cuttings. In the auxin response study, we compared high-nitrogen with low-nitrogen cuttings to investigate whether the different auxin response at the molecular level becomes manifested also in the rooting phenotype.

**Fig. 6.**
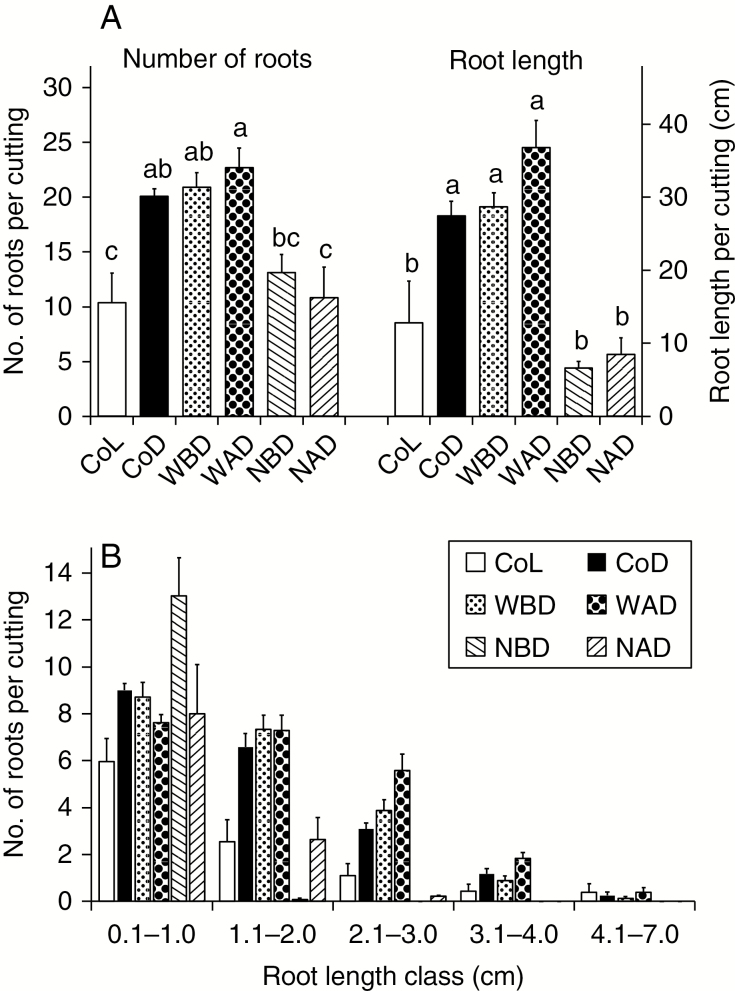
Effect of naphthylphthalamic acid (NPA) on dark-mediated AR formation in *Petunia hybrida*. Cuttings were either planted immediately and cultivated under light (Light treatment, CoL) or first dark incubated (10 °C) for 7 d and planted thereafter (Dark treatment, CoD). Other dark-treated cuttings were sprayed with water before (WBD) or after dark incubation (WAD), or with NPA (100 µm) before (NBD) or after dark incubation (NAD). (A) Number and total length of roots per planted cutting at day 16 post-excision of cuttings. (B) Distribution of roots above distinct root length classes. Mean values and s.e. of four independent biological replicates (*n*), each consisting of six cuttings. Columns which do not share a common letter are significantly different (*P* ≤ 0.05, ANOVA, Tukey’s HSD test).

As illustrated in Fig. 6A, dark incubation of cuttings strongly enhanced the number and length of ARs that were produced during 16 d after excision. By contrast, NPA strongly reduced AR formation and eliminated the benefit of dark incubation. Total AR number and length responded similarly to early and late NPA application (Fig. 6A), while only the early application of NPA restricted maximum root length to 1 cm (Fig. 6B).

**Fig. 7.**
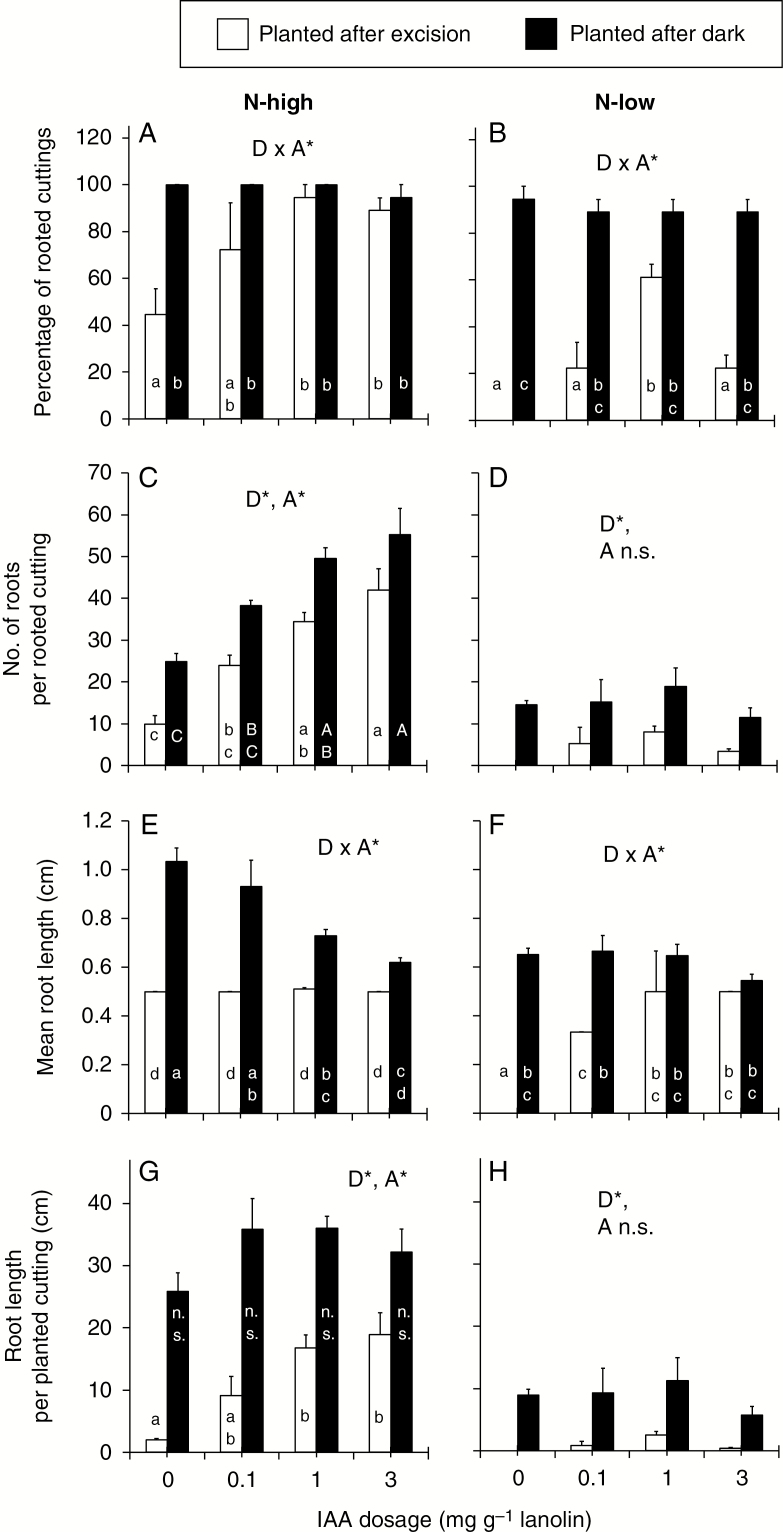
Effect of a pre-planting application of indole-3-acetic acid (IAA) until day 3 after planting on AR formation in *Petunia hybrida* as affected by nitrogen pre-conditioning and dark incubation of cuttings before planting. Lanolin paste containing IAA was applied to the cutting surface of the stem base. Cuttings were planted either immediately after excision or after dark incubation (7 d, 10 °C) and then cultivated in the light. Rooting was assessed at 9 d post-planting. Percentage of rooted cuttings of (A) high-N and (B) low-N cuttings, number of roots per rooted cutting of (C) high-N and (D) low-N cuttings, mean length per root of (E) high-N and (F) low-N cuttings, and total root length per planted cutting of (G) high-N and (H) low-N cuttings. D*, A* and (D x A*) indicate significant effects of dark incubation, of auxin and significant interaction between both factors, respectively. Columns which are indicated by letters and do not share a common letter are significantly different (*P* ≤ 0.05, ANOVA, Tukey’s HSD test, *n* = 3, each with six cuttings).

Since preliminary studies revealed negative side effects of petunia stem insertion into water, in the next experiment lanolin paste was used as a carrier of four different concentrations of IAA and applied to the stem base of cuttings over a period of 3 d. These treatments involved high- and low-nitrogen cuttings immediately after excision (planted after excision) or after being incubated in darkness for 7 d (planted after dark). With regard to AR formation in high-nitrogen cuttings, auxin application could substitute dark incubation to a great extent (Fig. 7). By contrast, the auxin response of AR formation was generally low in nitrogen-limited cuttings (Fig. 7).

The percentage of rooted cuttings was 40 % and 0 % for high- vs. low-nitrogen cuttings, when cuttings were planted immediately after excision, but increased in both cases to almost 100 % when cuttings experienced dark incubation before planting (Fig. 7A, B). Interestingly, this effect of dark exposure on high-nitrogen cuttings could be substituted by auxin while the medium IAA concentration (1 mg g^−1^ lanolin) already gained the same rooting percentage as the auxin-free dark-incubated cuttings (Fig. 7A). This auxin response was reduced in nitrogen-limited cuttings, which showed a maximum rooting percentage of 60 % after medium auxin supply (Fig. 7B). The number of ARs per rooted cutting was positively related to the applied auxin concentration in high-nitrogen cuttings, while the lowest auxin supply to non-dark-treated cuttings resulted in the same root number as the dark-incubated cuttings without auxin application (Fig. 7C). By contrast, numbers of ARs formed per rooted nitrogen-limited cutting generally remained below the respective values of high-nitrogen cuttings and did not show a significant auxin response (Fig. 7D). High-nitrogen cuttings were not affected by auxin in mean AR length when immediately planted but showed a decrease in AR length with increasing auxin concentration after dark incubation (Fig. 7E). With regard to low-nitrogen cuttings, auxin supply could partially substitute the positive effect of dark incubation on mean root length, whereas the generally longer ARs of dark-incubated cuttings did not show such an auxin response (Fig. 7F).

Total AR length per planted cutting integrates all processes from induction until elongation of ARs. The respective data indicate that auxin application substantially substituted the effect of dark exposure on the whole process of AR formation in high-nitrogen cuttings but almost completely failed in this function in nitrogen-limited cuttings (Fig. 7G, H). When high-nitrogen cuttings were planted after excision, application of auxin up to the highest concentration increased total root length from 8 % to 73 % of the level that was attained after additional dark incubation but without auxin application. By contrast, no significant auxin effect on this parameter was found in nitrogen-limited cuttings.

## DISCUSSION

Dark incubation of *P. hybrida* ‘Mitchell’ cuttings for 7 d at high (20 °C) or low temperature (10 °C) before planting enhances the number and length of ARs formed when compared to cuttings which are planted immediately after excision and experience a daily photosynthetic light integral of 3.6 mol m^2^ d^−1^ and a mean temperature of 21 °C (Klopotek *et al.*, 2010). Because dark incubation at the higher temperature causes stress to cuttings, as reflected by yellow and wilted leaves (Klopotek *et al.*, 2010), the present investigation focused on the hormonal mechanisms involved in the effective dark response of cuttings in combination with the lower temperature. The overall data provide evidence for an outstanding role of auxin homeostasis and signalling in dark-stimulated AR formation, while auxin action is further dependent on the nitrogen status of the cutting provided by the nitrogen nutrition of the stock plants.

### Dark incubation enhances the auxin signal during AR induction independent of nitrogen but dependent on the upper shoot

In the stem base of petunia cuttings, the first new cell clusters of developing ARs are observed until 72 hpe under light and until 144 hpe under dark conditions (Ahkami *et al.*, 2009; Klopotek *et al.*, 2010). Thus, the higher maximum concentrations at 4 and 6 hpe in the dark and the extension of the IAA peak until 72 hpe in the same tissue when compared with light-exposed cuttings (Fig. 1) provide a higher intensity and longer duration of the auxin signal in the rooting zone during the induction phase. In cuttings of carnation, efficient basipetal export of applied ^3^H-IAA from the two oldest leaves suggested their function as an auxin source while PAT in the stem sections was significantly lower at 4 °C than at 25 °C (Garrido *et al.*, 2002). In the present study, the elimination of IAA accumulation in the stem base of dark-exposed cuttings by their decapitating above the second oldest leaf (Fig. 2A) reveals the essential role of the upper shoot as a source of the extra IAA. The rapid increase of IAA in the stem base after IAA application to the apical cut end of the decapitated cuttings (Fig. 2B) highlights an efficient PAT under dark incubation even though temperature was reduced to 10 °C. Transcript levels of *PH-LAX2* and *Ph-PIN1* were not influenced by dark incubation before 24 hpe (Supplementary Data Table S2C). This suggests that the stronger early accumulation of IAA in the stem base of dark-exposed cuttings is not based on changed intensity of PAT, but rather on enhanced auxin mobilization in the upper shoot as an auxin source. Despite the lowered temperature, cuttings experience carbohydrate depletion during dark incubation resulting from the ongoing utilization of carbon which is not covered by photosynthesis. In petunia, this dark-induced carbohydrate depletion is more pronounced in leaves than in the stem base (Klopotek *et al.*, 2010) and the low energy status of cutting tissues triggers proteolysis that provokes a strong rise in amino acid concentrations (Zerche *et al.*, 2016). Although the concentration of l-tryptophan (Trp) as an important precursor of IAA was not analysed, the generally larger amino acid pool in dark-exposed cuttings may further involve increased availability of Trp that may contribute to enhanced IAA biosynthesis (Ljung, 2013). Processes downstream of Trp may also be stimulated by darkness. In this context, higher transcript levels of three YUCCA genes encoding flavin-containing monooxygenases that convert indole-3-pyruvate to IAA were found in dark-cultivated Arabidopsis leaf explants when compared to light conditions (Chen *et al.*, 2016).

### Dark incubation stimulates auxin signalling and function

The high number of up-regulated genes of the auxin pathway (Fig. 3) reflects a strong transcriptional stimulation of the action of auxin in the stem base by dark incubation of cuttings. Because detailed temporal analysis of 12 selected genes of different functional groups did not indicate a dark response until 6 hpe (Supplementary Data Table S2C), the early increase in auxin concentration might have induced many of these responses. Positive feedback loops are well established between auxin level and transcription of *PIN1* (Omelyanchuk *et al.*, 2016). *PIN1* has an important function during AR formation in Arabidopsis, where it mediates auxin stimulus via provision of a lateral auxin efflux from the vasculature towards the AR founder cells (Della Rovere *et al.*, 2013; Sukumar *et al.*, 2013). Thus, up-regulation of *Ph-PIN1* (Fig. 3A, Supplementary Data Table S2C) might be triggered by the dark-induced rise in IAA concentration (Fig. 1) and contribute to the dark-promoted AR induction in petunia cuttings (Fig. 7A, B). In Arabidopsis, *LAX3* shows high expression particularly during later stages of AR formation, clearly contributing to auxin maximization in the AR tip (Della Rovere *et al.*, 2013). In light of these findings, the down-regulation of *Ph-LAX2* and *Ph-LAX3* in petunia during the dark phase (Fig. 3A) and the corresponding transient repression of *Ph-CYCB1* (Supplementary Data Table S2C), which putatively encodes an auxin-responsive cyclin-dependent kinase as an important regulator of the cell cycle (Hartig and Beck, 2006; Komaki and Sugimoto, 2012), agrees with the phenomenon that dark incubation slows down the current speed of AR initiation, but provides a ‘pole position’ for accelerated AR development during the post-dark phase (Klopotek *et al.*, 2010).

Dark-induced down-regulation of *Ph-GH3.1* and *Ph-GH3.3* at 24 hpe and up-regulation of *Ph-GH3.6* and *Ph-GH3.10* at 72 hpe (Figs 3A and 4A, B) suggest different functions of the specific GH3 proteins during dark-mediated AR formation in petunia. One important function of auxin-induced GH3 proteins is their activity as acyl acid amido synthetases while several isoenzymes in Arabidopsis have already been characterized to conjugate IAA, jasmonic acid (JA) or salicylic acid, or may even have multiple functions (Staswick *et al.*, 2005; Westfall *et al.*, 2016).

Molecular analysis of Arabidopsis mutants indicated that *GH3.3*, *GH3.5* and *GH3.6* control each other’s expression and have positive functions during etiolation-induced AR formation in intact seedlings (Gutierrez *et al*., 2009, 2012). The authors postulated a model in which the three GH3 proteins inactivate JA, which in is Arabidopsis inhibitory to AR initiation, through formation of inactive amino acid conjugates in competition with the GH3.11-mediated generation of physiologically active jasmonyl-l-isoleucine. In a recent study on carnation, however, higher transcript levels of *DcGH3.1* in the stem base of a poorly rooting carnation cultivar during the induction phase corresponded to higher concentrations of IAA-Asp at the expense of free IAA when compared to the good-rooting cultivar, whereas chemical blocking of GH3 enzymatic activity reduced the deficit in AR formation (Cano *et al.*, 2018). Regarding these findings, the dark-reduced expression of *Ph-GH3.1* in petunia (Fig. 3A) may contribute to AR induction via extension of the IAA peak (Fig. 1A).

The dark-stimulated expression of *Ph-ARF8* and *Ph-ARF10* in nitrogen-unlimited cuttings (Figs 3B and 5C, D) indicates important roles of the corresponding proteins as positive regulators during dark-mediated AR induction in petunia cuttings. ARF8 is a positive regulator of etiolation-induced AR formation in hypocotyls of intact Arabidopsis seedlings (Gutierrez *et al.*, 2012). In far-red light-acclimated cuttings of *Eucalyptus globulus*, higher expression of *ARF8* during induction and later rooting stages was correlated with enhanced AR formation when compared with white light-acclimated cuttings (Ruedell *et al.*, 2015). In black walnut (*Juglans nigra* L.), auxin induced the expression of *ARF8* specifically in phloem parenchyma cells of rooting-competent cuttings before AR primordia were formed (Stevens *et al.*, 2018).

Considering the function of Aux/IAA proteins as transcription repressors of *ARFs*, the dark-induced up-regulation of *Ph-IAA4*, *Ph-IAA7*, *Ph-IAA14*, *Ph-IAA17* and *Ph-IAA19* at 24 hpe and/or 72 hpe in the present study appears inconsistent with the simultaneous up-regulation of several *ARFs* (Fig. 3B). However, enhanced transcription of *Aux*/*IAA* genes may be the consequence of previous degradation of Aux/IAA proteins (Krogan and Berleth, 2015). For example, in Arabidopsis the release of *ARF5* from repression by auxin-induced degradation of IAA1, IAA19 and IAA20 stimulates the expression of several *Aux*/*IAA* genes including those encoding the degraded Aux/IAA proteins (Krogan *et al.*, 2014; Krogan and Berleth, 2015). In light of these relationships, the higher transcript levels of the five *Aux*/*IAA* genes in dark-exposed cuttings probably reflect earlier auxin-induced degradation of respective or other Aux/IAA proteins that form regulatory modules with the dark-induced *ARFs*. According to this scenario, an enhanced rooting response of juvenile cuttings of *Eucalyptus grandis* to auxin application was correlated with stronger *IAA19* expression (Abu-Abied *et al.*, 2014). Furthermore, enhanced AR formation of a specific carnation cultivar was correlated with higher IAA concentrations and higher transcript levels of *DcIAA3*, *DcIAA4*, *DcIAA13* and *DcIAA19* in the stem base (Cano *et al.*, 2018). The identity and function of distinct Aux/IAA-ARF modules during AR formation are rarely understood. Recent analysis of Arabidopsis gain-of-function mutants of specific Aux/IAA genes and a double loss-of-function mutant of *ARF7* and *ARF19* revealed that AR formation in leaf explants is inhibited when the functions of *ARF7* and *ARF19* are both impaired while the results further indicated that IAA18, IAA14 and IAA28 proteins are required to mediate auxin signalling during vascular proliferation, AR initiation and during both processes, respectively (Bustillo-Avendaño *et al.*, 2018). In another recent study of AR formation in hypocotyls of intact Arabidopsis seedlings, analysis of knock-out mutants of *IAA6*, *IAA9* and *IAA17* highlighted that the encoded proteins function as auxin repressors via down-regulation of ARF6 and/or ARF8, which act as positive regulators of etiolation-induced AR formation (Lakehal *et al.*, 2019).

SAUR proteins are known to be transcriptionally induced by auxin in diverse plant species and are involved in hormone-mediated plant development (Ren and Gray, 2015). However, auxin-induced down-regulation of SAUR genes has also been observed particularly in roots of Arabidopsis (Paponov *et al.*, 2008). Information regarding the roles of SAURs in primary and lateral root development is fragmentary and conflicting, while their roles in AR formation are unknown (Kant *et al.*, 2009; Kong *et al.*, 2013; Markakis *et al.*, 2013). Nevertheless, the broad dark response of genes of the *SAUR* family showing preferential upregulation (eight of 11 genes) but also down-regulation of distinct genes (Figs 3D and 4C, D) points towards important functions during dark-mediated AR formation. In shoots and primary roots, specific SAURs act dependent on the TIR1-Aux/IAA machinery and control cell expansion via targeting PP2C.D phosphatases, which function as inhibitors of plasma membrane H^+^-ATPases, while the resulting acidification of the apoplast is thought to activate expansins (Spartz *et al.*, 2014; Ren and Gray, 2015; Fendrych *et al.*, 2016; Ren *et al.*, 2018). In the present study, dark incubation induced the expression of many genes putatively encoding expansins at 24 and 72 hpe (Supplementary Data Table S2D). Thus, a function of *SAURs* similar to that in shoot growth may be involved in dark-mediated AR induction.

### PAT and enhanced IAA concentration in the stem base contribute to dark-mediated AR formation

Elimination of the dark-mediated increase in AR number and length by application of NPA (Fig. 6A) clearly demonstrates the essential role of PAT in dark stimulation of AR formation in petunia. The additional finding that NPA application before dark incubation restricted the length of all formed ARs below 1 cm (Fig. 6B) can be explained by major inhibition of AR induction as an early bottleneck for AR formation. By contrast, NPA application after dark only impaired, but did not prevent, the formation of longer roots (Fig. 6B). This indicates that the late NPA application mostly interfered with differentiation and growth during initiation, expression and elongation of ARs that occurs after dark incubation (Klopotek *et al.*, 2010).

The auxin response of rooting was in line with the coherent picture of dark-mediated auxin homeostasis and signalling. When nitrogen was not limited, basal application of IAA to light-exposed cuttings during the induction phase (until 3 dpe) could mimic to a great extent the enhanced IAA accumulation in dark-incubated cuttings in terms of AR formation (Fig. 7A, C, E, F). These findings strongly support the functional role of the dark-mediated greater auxin signal in dark-stimulated rooting. The complete substitution of the effect of dark exposure on the percentage of rooted cuttings by IAA application (Fig. 7A) highlights the AR inductive role of dark-mediated IAA accumulation (Fig. 1). The higher number of ARs per rooted cutting in response to IAA and to dark can be explained by a higher number of induced cells per stem base that developed into AR initials (Fig. 7C). While the lowest IAA dosage applied to the non-dark-incubated cuttings was sufficient to mimic the effect of dark exposure, the positive auxin response also of the dark-exposed cuttings can be explained by the condition that root induction is still not completed after 7 d of dark incubation (Klopotek *et al.*, 2010). According to the inhibitory role of high auxin concentrations on root elongation (da Costa *et al.*, 2013), auxin application reduced the mean root length of dark-incubated cuttings (Fig. 7E), which were already in the AR initiation and emergence phases when auxin was applied (Klopotek *et al.*, 2010). Together, these results suggest that the enhanced auxin signal substantially accounts for the dark-stimulated AR formation in nitrogen-unlimited cuttings.

### Nitrogen limitation inhibits the auxin response at the molecular and root phenotype level

Transcript levels of genes related to auxin revealed a decreased auxin signalling in nitrogen-limited cuttings at the levels of *Ph-GH3.6*, *Ph-GH3.10*, *Ph-IAA14*, *Ph-ARF8*, *Ph-ARF10* and *Ph-SAUR14* expression from 24 hpe until 48 hpe particularly under dark incubation (Figs 4 and 5A, C, D). Nitrogen limitation further reduced the dark-induced down-regulation of *Ph-SAUR14*. Even though the functions of these components have not been analysed in petunia, the expression data support an inhibitory role of N limitation on auxin signalling and function during AR induction.

The reduced auxin response of AR formation in N-limited cuttings confirms the inhibitory role of the reduced molecular auxin signalling for the root phenotype (Fig. 7). The strongly reduced total root length per planted cutting compared with high-nitrogen cuttings and the missing auxin response of this parameter (Fig. 7H) indicates that AR formation is limited by factors downstream or even independent of the auxin signal. The weaker auxin response of the percentage of rooted cuttings compared with high-nitrogen cuttings (Fig. 7A, B) and the missing auxin response of the number of ARs per rooted cutting in N-limited cuttings (Fig. 7D) indicates that at the level of individual cuttings, no or only very few cells in the stem base were able to form ARs in response to auxin. Interestingly, an earlier study under similar environmental conditions revealed that N limitation specifically inhibits the differentiation of new cell clusters into AR primordia (Zerche *et al.*, 2016). The molecular and root phenotype data of the present study indicate that N limitation impairs auxin signalling already during the induction phase, which impedes the reprogramming of potential AR source cells. The positive effect of auxin application on the mean root length of non-dark-exposed cuttings (Fig. 7F) does not reflect a stimulation of root elongation, which would contradict the accepted inhibitory role of auxin on this process (da Costa *et al.*, 2013), but is based on the fact that the auxin-free cuttings did not produce any root (Fig. 7D).

We did not analyse concentrations of Ck, which can have antagonistic effects to auxin during AR induction (Druege *et al.*, 2019). Ck concentrations are usually lower in tissues from nitrogen-limited plants when compared to plants that receive adequate nitrogen supply (Sakakibara *et al.*, 2006; Kiba *et al.*, 2011), which would not explain the weaker auxin response in nitrogen-limited cuttings found in the present study. However, there are further indications in the literature that N availability can limit auxin signalling via the TIR1/AFB-Aux/IAA-ARF complex and may further involve microRNAs (Vidal *et al.*, 2010; Kiba *et al.*, 2011; Krouk, 2016). Arginine is assumed as a putative donor of nitric oxide in plants, which has a positive function in auxin signalling during AR formation probably involving TIR1-mediated degradation of Aux/IAA proteins (Druege *et al.*, 2016). The auxin receptor gene *AFB3* in Arabidopsis is induced by nitrate but thereafter is repressed by metabolites downstream of nitrate such as ammonium and glutamate dependent on miR393 (Vidal *et al.*, 2010). Most interestingly, Gifford *et al.* (2008) demonstrated that inhibited emergence of lateral roots in Arabidopsis by high nitrogen supply is mediated by glutamine and involves enhanced expression of *ARF8* in root pericycle via release from the repression by miR167 as well as enhanced transcription of several *Aux/IAA* genes. miR167 also has a function in the control of *ARF8* during etiolation-induced AR formation in Arabidopsis (Gutierrez *et al.*, 2009). Furthermore, nitrogen starvation enhanced the expression of miR160 and correspondingly reduced the expression of its target gene *ARF10* in Arabidopsis (Liang *et al.*, 2015). Recently, Zerche *et al.* (2016) showed that dark incubation enhances the concentrations of arginine and glutamine in leaves and the stem base of nitrogen-limited cuttings, respectively, but the levels attained are still below those attained in high-nitrogen cuttings. Thus, with regard to the mechanisms discussed above, suboptimal concentrations of amino acids, particularly of glutamine and arginine, in the nitrogen-deficient cuttings might have contributed to the reduced auxin signalling and reduced auxin response at the transcriptional and root phenotype level. Given that the target of rapamycin (TOR) kinase (which integrates nutrient sensing, for example via glutamine with auxin signaling) has recently been shown to control AR formation in potato and Arabidopsis via the TIR1 auxin receptor (Dobrenel *et al.*, 2016; Deng *et al.*, 2017), TOR may provide a node integrating nitrogen metabolism with auxin signalling in petunia.

### Auxin–metabolite interactions on dark-mediated AR formation

**Fig. 8.**
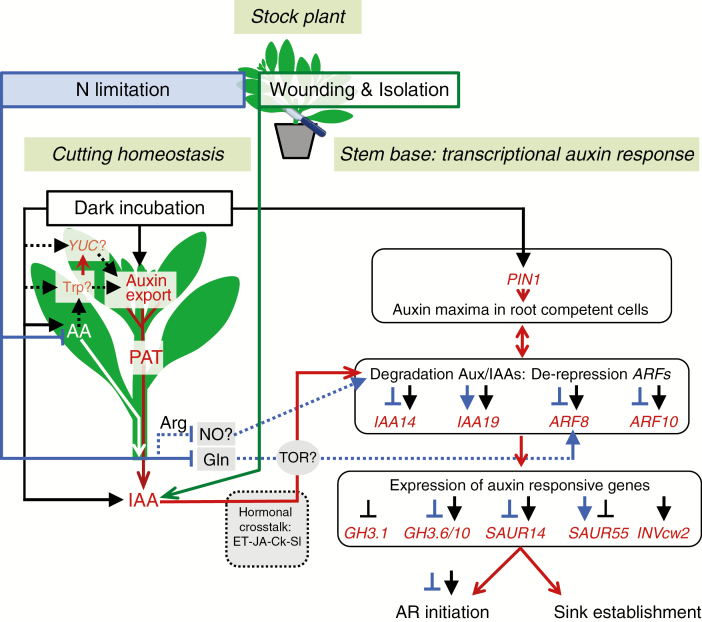
Working model of auxin function during nitrogen and dark-mediated AR formation in *Petunia hybrida* cuttings. Red arrows indicate the direction of PAT and the pathway of auxin action, while auxin-related processes and factors are indicated by red letters. White arrows indicate the transport of amino acids. Blue and black arrows, ending with arrowheads vs. crossbars indicate promotive vs. inhibitory influences of nitrogen limitation and dark incubation, respectively. Broken lines indicate hypothetical relationships based on further literature discussed in the text. AA, amino acids; Arg, arginine; Gln, glutamine; NO, nitric oxide; IAA, indole-3-acetic acid; ET, ethylene; JA, jasmonic acid; Ck, cytokinins; Sl, strigolactones; PAT, polar auxin transport; Trp, l-tryptophan; YUC, YUCCA; TOR, target of rapamycin kinase.

Based on the current data and former studies on the anatomical and metabolic response of *P. hybrida* to dark incubation and nitrogen pre-conditioning of cuttings (Klopotek *et al*., 2010, 2016; Zerche *et al.*, 2016), a working model for the function of auxin in dark-promoted AR formation in petunia is illustrated in Fig. 8. Wounding and isolation of cuttings from the stock plant initiate an early accumulation of IAA in the rooting zone, while IAA accumulation is to great extent the result of a PAT-dependent auxin influx as additionally supported by local wound-induced mobilization of IAA conjugates (Druege *et al.*, 2014). Dark incubation of cuttings enhances the maximum concentrations and extends the IAA peak in the stem base during AR induction. The additional IAA is delivered from the upper shoot, while the auxin source capacity of the upper shoot is possibly enhanced through proteolysis-driven enhanced availability of the IAA precursor Trp and/or up-regulation of YUCCA genes. Probably involving crosstalk with ethylene, JA, Ck and strigolactones (Druege and Franken, 2019), the increased IAA concentration enforces the auxin signalling cascade via enhanced degradation of Aux/IAA proteins, such as Ph-IAA14 and Ph-IAA19, so that specific ARF-encoding genes (e.g. *Ph-ARF8* and *Ph-AR10*) are released from repression. The resulting abundant ARF proteins enhance the transcriptional response of auxin responsive proteins including Ph-PIN1 that promotes the establishment of auxin maxima in root competent cells. Changed transcription of *GH3* genes, particularly of *Ph-GH3.1*, *Ph-GH3.6* and *Ph-GH3.10*, as well as enhanced expression of a large set of SAUR genes including *Ph-SAUR14* vs. distinct down-regulation of specific SAUR genes such as *Ph-SAUR55* control the initiation of ARs, possibly via conjugation of IAA or JA and by control of cell expansion. In addition, auxin-induced up-regulation of *Ph-INVcw2* (Ahkami *et al.*, 2013) contributes to sink establishment. Nitrogen limitation in the cuttings has only marginal effects on IAA concentration in the stem base but attenuates auxin signalling at the expression levels of specific *ARFs*, *GH3* genes and *SAUR* genes, so that the auxin dose–response of AR initiation is suppressed. This may result from the reduced concentrations of amino acids, particularly of arginine as a possible donor of NO and of glutamine as a positive regulator of *ARF8*, while TOR may function as a node of nutrient–auxin integration. On the other hand, dark incubation enhances the concentrations of these amino acids, which may trigger auxin signalling via these mechanisms.

Based on this working model, future studies should focus in particular on the molecular and physiological mechanisms of dark-mediated auxin mobilization from the upper shoot and the functional roles of the specific *Aux*/*IAA*, *ARF* and *SAUR* genes in AR formation in cuttings. The available genome sequences of two parental lines of *P. hybrida* (Bombarely *et al.*, 2016) and the other tools available in the petunia scientific community for the functional analysis of genes (Druege and Franken, 2019) provide good perspectives for future detailed investigations of regulatory modules controlling nitrogen- and light-mediated AR formation in cuttings.

## SUPPLEMENTARY DATA

Supplementary data are available online at https://academic.oup.com/aob and consist of the following. Table S1: Primers used to analyse the transcript levels of genes controlling auxin homeostasis, signalling and function in *Petunia hybrida*. Table S2: A, mean expression values of auxin-related genes in the stem base of petunia either cultivated under light or dark, as revealed by microarray analysis; B. dark/light expression ratios for selected auxin-related genes as revealed by microarray analysis; C, transcript levels of selected auxin-related genes relative to the reference gene *Ph-RPS13*, as revealed by qRT-PCR; D, dark/light expression ratios for expansin-like genes as revealed by microarray analysis.

## FUNDING

This work was supported by the Deutsche Forschungs-gemeinschaft (DR 411/2-1) and by the States of Brandenburg, the Free State of Thuringia and the Federal Republic of Germany.

## Supplementary Material

mcz095_suppl_Supplementary_Table_S1

mcz095_suppl_Supplementary_Table_S2
