# Supplementary material for: Role of auxin homeostasis and response in nitrogen limitation and dark stimulation of adventitious root formation in petunia cuttings
Source: Ann Bot. 2019 Jun 10;124(6):1053–66. doi: 10.1093/aob/mcz095 (PMC6881223; doi:10.1093/aob/mcz095)
Supplement: mcz095_suppl_Supplementary_Table_S1 [file mcz095_suppl_supplementary_table_s1.docx]

**Table S1** Primers used to analyze the transcript levels of genes controlling auxin homeostasis, signalling and function in *Petunia hybrida*

| Gene | Forward primer (5’->3’) | Reverse primer (5’->3’) |
| --- | --- | --- |
| *Ph-PIN1* | TCAATGGTTGCTGGTGGTAG | TTTGAAGGCCTAGGAGTTGG |
| *Ph-LAX2* | GAGATAATGCACGCGATGTG | TAAACTGCTGCTGCTGATGG |
| *Ph-GH3.6* | AAAGATGTCCTCCGTGTTGG | TGACAAAGAAGAGGCCAGTG |
| *Ph-GH3.10* | ATGGGTCCACAGAGAGTTGG | CGGTGGCTAGAATTGCATCT |
| *Ph-IAA14* | CAAAAAGGACTCAGCCAAGC | GCACCATCCATTGAGACCTT |
| *Ph-IAA19* | GCGACGGAGATCAAGAAAAG | ACCCCACAACTTGGTTCTTG |
| *Ph-ARF5* | ATCCAGGCACAATTGGAGTC | TTCAGCAGCAGATTGGACAG |
| *Ph-ARF8* | TGGGTAGCTGGGAAAATGAG | ATTTCCCTCAAGGGCATAGC |
| *Ph-ARF10* | GCAGCTGATTCCAAACTTCC | ACCCATGGTAAGCAAGCAAG |
| *Ph-SAUR14* | TTCACAGCAGGGAATTGTGA | TATTCGTGTGCCTCGTATGC |
| *Ph-SAUR55* | GGCCTCAGTTCTGCTCACTC | AACCAATGGTCCTTCACTGC |
| *Ph-CYCB1* | GGTTACACGTCGTGGTGTTG | TCTGAGCTGCAGGTTTCCTT |
| *Ph-RPS13* | AAGCTCCCACCTGTCTGGAAA | AACAGATTGCCGGAAGCCA |
